# Myb Immunohistochemical Staining and Fluorescence *in situ* Hybridization in Salivary Rare Basaloid Lesions

**DOI:** 10.3389/fonc.2020.00870

**Published:** 2020-06-30

**Authors:** Binbin Li, Weiping Jie, Huiying He

**Affiliations:** ^1^Department of Oral Pathology, Peking University School and Hospital of Stomatology, Beijing, China; ^2^Research Unit of Precision Pathologic Diagnosis in Tumors of the Oral and Maxillofacial Regions, Chinese Academy of Medical Sciences, Beijing, China; ^3^National Clinical Research Center for Oral Diseases, Peking University School and Hospital of Stomatology, Beijing, China; ^4^Department of Pathology, School of Basic Medical Sciences, Third Hospital, Peking University Health Science Center, Beijing, China

**Keywords:** MYB, immunohistochemistry, fluorescence *in situ* hybridization, salivary basaloid lesions, adenoid cystic carcinoma

## Abstract

**Objective:** Salivary rare basaloid lesions, including cribriform type basal cell adenoma (cBCA), BCA with incomplete capsule (iBCA), sialoblastoma (SB), and intercalated duct hyperplasia (IDH), could easily be misdiagnosed as adenoid cystic carcinoma (AdCC). We aim to identify an approach for differential diagnosis and to establish an optimal workflow concerning the diagnosis of these lesions.

**Material and methods:** A panel of antibodies (MYB, β-catenin, CD117, SOX10, ki67, P63, calponin) and fluorescence *in situ* hybridization (FISH)-MYB were utilized to distinguish above salivary basaloid diseases from AdCC.

**Results:** Histologically, the striking diagnostic features of cBCA, iBCA, SB, and IDH are composed of basaloid tumor cells, well-defined encapsulation, or lack of destructive invasion. Immunohistochemically, Myb immune-labeling could effectively make a distinction among cBCA, iBCA, SB, and IDH from AdCC, except in SB. cBCA and iBCA typically expressed β-catenin in the nuclei of tumor cells. There was no statistical significance in the ki67 index between SB and AdCC, but their indices were significantly higher than those of iBCA and IDH (*p* < 0.05, *p* < 0.05, respectively). P63 and calponin immune-expression were observed in the basaloid or myoepithelial cells. CD117 were observed positively in cBCA, iBCA, SB, and AdCC, except in IDH. SOX10 were observed positively in all cases. No cases had fusion of MYB and NFIB detectable by FISH, except in AdCC.

**Conclusion:** Considering their sensitivity and specificity, FISH-Myb and an immunohistochemical panel of MYB/β-catenin/ki67 would be an optimal choice for the differential diagnosis of these basaloid lesions.

**Clinical relevance:** Some salivary basaloid tumor or tumor-like lesions have overlapping features with AdCC. Through this present research, we suggested that the panel IHC of MYB, βcatenin, and ki67 combined with FISH-Myb should be an optimal choice for differential diagnosis among those lesions.

## Introduction

Salivary rare basaloid tumor or tumor-like lesions share some features resembling adenoid cystic carcinoma (AdCC), even exhibiting a typical cribriform pattern like that of AdCC. This creates diagnosis pitfalls, especially in fine-needle aspiration testing (FNAT). iBCA and cBCA are usually treated with a conservative treatment strategy (partial or superficial parotidectomy), which is very different from the radical choice of AdCC (radical surgical excision with or without postoperative radiation). No surgical intervention is required for IDH. Most SBs are cured by primary surgical resection. Therefore, it is very important to make a differential diagnosis between them. In addition, these lesions may be less familiar to non-head and neck specialists due to the low incidence. Since exact diagnosis is crucial for an appropriate treatment choice, it is necessary to study the following AdCC mimicking lesions using the specific protein and molecular markers.

Usually, typical basal cell adenoma (BCA) is well-circumscribed and composed of basaloid cells with eosinophilic cytoplasm (1). In practice, there are two types of BCA, cribriform BCA (cBCA) and BCA with incomplete encapsulation (iBCA), which are easily misdiagnosed as AdCC. Because AdCC is mainly characterized by a cribriform growth pattern, the cribriform components apparent in cBCA would mislead pathologists to the diagnosis of AdCC. Our primary study and a recent study both proved that AdCC and cBCA were two distinct tumor entities (2, 3). Some studies suggested that β-catenin mutation was present in up to 52% of BCAs. Thus, the corresponding nuclear expression of β-catenin can be detected in BCA and would be a specific marker to identify cBCA or iBCA from AdCC (4). At the same time, few BCAs have incomplete capsules or could have focal capsule invasion, which are easily mistaken as malignant presentation. In this circumstance it is important to differentiate iBCA from AdCC, despite the capsule of micro-invasion.

Apart from iBCA and cBCA, sialoblastoma (SB), and intercalated duct hyperplasia (IDH) could occasionally resemble AdCC too. SB was ever named as congenital hybrid basal cell adenoma-adenoid cystic carcinoma (5), which suggested that its morphology overlapped with BCA or AdCC. In some instances the cribriform pattern was evident in SB, and it was problematic to distinguish between them. IDH is a salivary ductal proliferation resembling intercalated ducts, which was newly identified as a separate entity in the WHO new classification of Head and Neck Tumors 2017. It is considered as a reactive and hyperplastic process, or a precursor condition for some salivary gland tumors, such as BCA (6). It typically exhibited an idiopathic, focal hypertrophic lesion of intraoral mucous glands with limited growth possibilities (7). Due to its unencapsulation, IDH may also be misdiagnosed as AdCC.

Recent genomic study identified new markers that may be helpful in future investigations. Researchers have shown that the specific translocation *t* (6;9) involving v-myb avian myeloblastosis viral oncogene homolog (MYB) and nuclear factor 1B-type (NFIB) was the most relevant genetic alternation in AdCC (8). MYB immuno-staining was proved to be a useful ancillary test for distinguishing AdCC from other benign and malignant salivary gland neoplasms (9, 10). On the other hand, the transcriptional factor sex-determining region Y (SRY)-related HMG box-containing factor 10 (SOX10), normally only expressed during salivary gland differentiation, was found markedly upregulated in a majority of AdCC cases (11). In addition, it was reported that most AdCCs (90%) exhibited strong and diffuse expression of CD117 (12). Thus, a panel of specific immunomarkers that include MYB, SOX-10, and CD117 could be believed as very effective for the diagnosis of AdCC.

Of course, the typical AdCC is generally recognized by biopsy in HE staining. However, iBCA, cBCA, SB, and IDH are extremely rare lesions, and are much more confusing in microscopic diagnosis. Based on above research progress, genetic abnormalities, and protein expressions may be helpful in their distinction from AdCC. Taken together, it is worth identifying an approach with relatively high sensitivity and high specificity in the histologic diagnosis of salivary basaloid lesions. This study first used a combination of histologic, immunohistochemical, and a FISH test to evaluate MYB, CD117, SOX10, βcatenin, ki67, p63, and calponin in the differential diagnosis of cBCA, iBCA, SB, and IDH from AdCC. It was shown that both SB and AdCC had MYB positive immune-expression, but only AdCC had Myb rearrangement examined by FISH. The positive reaction ofβ-catenin in nuclear was specific in iBCA and cBCA. P63 and calponin immune-expression were observed in the basaloid or myoepithelial cells. CD117 were observed positively in cBCA, iBCA, SB, and AdCC, except in IDH. SOX10 were observed positively in all the cases. The ki67 indices of SB and AdCC were significantly higher than those of iBCA and IDH. Taking account of both sensitivity and specificity, we recommend FISH-Myb and an immunohistochemical panel of MYB/β-catenin/ki67 as an optimal choice for the differential diagnosis of these basaloid lesions.

## Materials and Methods

### Patients

Approved by Institutional Review Board and formally numbered PKUSSIRB-201631121, 64 cases, including 23 cBCAs, 11 iBCAs, 3 SBs, 7 IDHs, and 20 AdCCs (as controls), were collected from the files of Peking University School and Hospital of Stomatology. The criteria for eligibility of AdCC were (i) histologically typical AdCC confirmed by two pathologists (Dr. Li and Dr. He) (ii) presence of adjacent tissue invasion, including muscle, gland, vessel, nerve, and bone infiltration or lung metastasis.

The investigated parameters consisted of gender, age distributions, anatomic location, histologic characteristics, treatment, and postoperative outcomes. Follow-up information was obtained from the medical records or by telephone interview.

### Histological Examination

All specimens were fixed in neutral buffered 10% formalin, processed and embedded in paraffin. 4 μm sections were stained with hematoxylin and eosin, and then observed under light microscope.

### Immunohistochemical Reaction

All slides were performed with the following seven antibodies by the standard streptavidin-biotin-peroxidase complex technique. The MYB antibody was bought from ABCAM Co. (clone EP769Y, dilution 1:500, ABCAM, Cambridge, MA). CD117 (clone EP10), SOX10 (clone EP268), βcatenin (clone UMAB15), ki67(clone UMAB107), p63 (clone 4A4), and calponin (clone EP63) were obtained from Zymed Co. (Zymed, Carlsbad, CA). The staining was scored qualitatively and assessed on intensity (1+ to 3+) and staining pattern (nuclear, cytoplasm or membrane). Only 2+ or 3+ intensity staining was interpreted as positive.

### FISH Test

A commercially available Myb dual-color break-apart probe (ZTV-Z-2143-200, ZytoVision, Bremerhaven, Germany) was utilized for FISH. Before hybridization, 4 μm slides were baked at 65°C overnight, deparaffinized by washing with xylene and ethanol, and rehydrated with deionized water at 90°C for 30 min. Pretreated slides were incubated with a proteinase K solution (100 mg/mL; Sigma-Aldrich, UK) for 10–30 min. Slides and probes were then co-denatured at 73°C for 5 min and hybridized overnight at 37°C using an automated ThermoBrite codenaturation oven (Abbott Molecular) according to the manufacturer's instructions. After hybridization, slides were immersed in a post-hybridization solution, 2xSSC/0.3% NP40 at 73°C for 2 min, and subsequently in 2xSSC/0.1%NP40 at room temperature for 1 min. Slides were dehydrated, dried, and counterstained with 4,6-diamino-2-phenylindole (DAPI; Vysis, USA).

All the FISH slides were reviewed and confirmed by two pathologists, and microscopically scored, respectively.

### Statistical Analysis

SPSS 17.0 software was used to perform statistical analysis and draw diagrams. Statistical comparisons of ki67 between all the groups were made using analysis of variance (ANOVA). The LSD *post-doc* assay was used when equal variance was assumed. Otherwise, Dunnetts T3 test was used. Correlation analysis was used to analyze the relation between MYB immuno-analysis and chromosome rearrangement. Differences of *P* < 0.05 were considered significant.

## Results

### Clinical Features

For all the 44 cases mimicking AdCC, the mean age was 45 years and the male/female ratio was 12/32. Most iBCAs (95%) occurred in the parotid glands. All cBCA and SB occurred in the parotid gland.

All the selected benign salivary diseases have a slow growth process and were well-demarcated. Localized swelling was the main symptom in all cases. All patients underwent complete surgical excision of these lesions and there had been no recurrence at the time when this study was written up (Dec. 2018). The median follow-up time was 87 months.

In particular, three cases of SB were infants or children, including two cases in 1-year-olds, and one case in a 12-years-old. The duration of follow-up for these three patients was 80, 119, and 92 months, respectively (Table 1).

**Table 1:** Clinical characteristics of all the basaloid lesions.

**Tumor**	**Median age (years)**	**Sex (Female/male)**	**Locations**	**Current status**	**Median follow-up duration (months)**
cBCA (*n* = 23)	48	16/7	parotid	Alive/No recurrence	110
iBCA (*n* = 11)	47	9/2	parotid	Alive/No recurrence	43
SB (*n* = 3)	5	1/2	parotid	Alive/No recurrence	97
IDH (*n* = 7)	48	6/1	Parotid5/submandibular2	Alive/No recurrence	75
Total (*n* = 44)	45	32/12	Parotid (95%)	Alive/No recurrence	87

### Histologic Findings

Generally, all the tumor types included in this study were composed mainly of basal-like cells. These small-to-medium basaloid cells consisted of a series of columnar, cuboidal, or polygonal cells. Cells had hyperchromatic nuclei with little cytoplasm, similar to the basal cells in stratified squamous epithelium. Although these basaloid cells shared similar cellular morphology, the tumors demonstrated different histologic patterns, immunoprofiles, and specific disease behavior.

**Figure 1:**
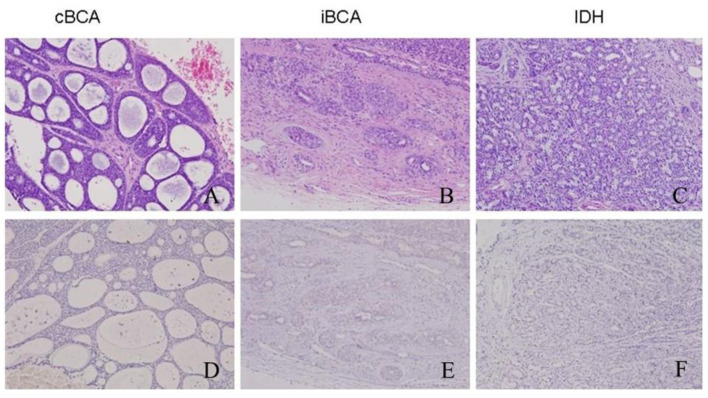
Histology (upper line) and MYB immune-expression (lower line) in cBCA, iBCA, and IDH. cBCA was well-defined and had cribriform patterns with pseudocyst formation **(A)**. Both iBCA and IDH had no definite capsule and presented no clear boundary between the tumor mass and the surrounding tissues **(B,C)**. But iBCA showed typical BCA characteristics of peripheral palisading in parenchyma. No immunoreactivity for MYB antigen was found in these three lesions **(D–F)**.

At low magnification, cBCA was well-demarcated and uniform in parenchyma. These tumors had cribriform patterns with pseudocyst formation containing amorphous and basophilic material, resembling AdCC (Figure 1A). At higher magnification, the outermost layers of cells surrounding each cell nest were usually cuboidal with peripheral palisading. Although both BCA and AdCC are thought to arise from the same basal stem cell, BCA had a more distinct pattern with micro-encapsulated areas that were not apparent in AdCC. BCA also displayed a clear separation of epithelium from stroma, which was never seen in AdCC.

For iBCA, the tumor cells may invade the capsule, or there may be no clear boundary between the tumor mass and normal glands (Figure 1B). Sometimes, the mass had no definite capsule, but was surrounded by a fibrous pseudocapsule. There existed a limited extension of tumor into normal glandular parenchyma, but this was not a sign of malignancy.

IDH was often accompanied by other benign or malignant lesions. It was usually ill-defined, and lacked destructive invasion, anaplasia, or mitotic activity, and the lobular pattern of normal salivary glands was preserved (Figure 1C).

**Figure 2:**
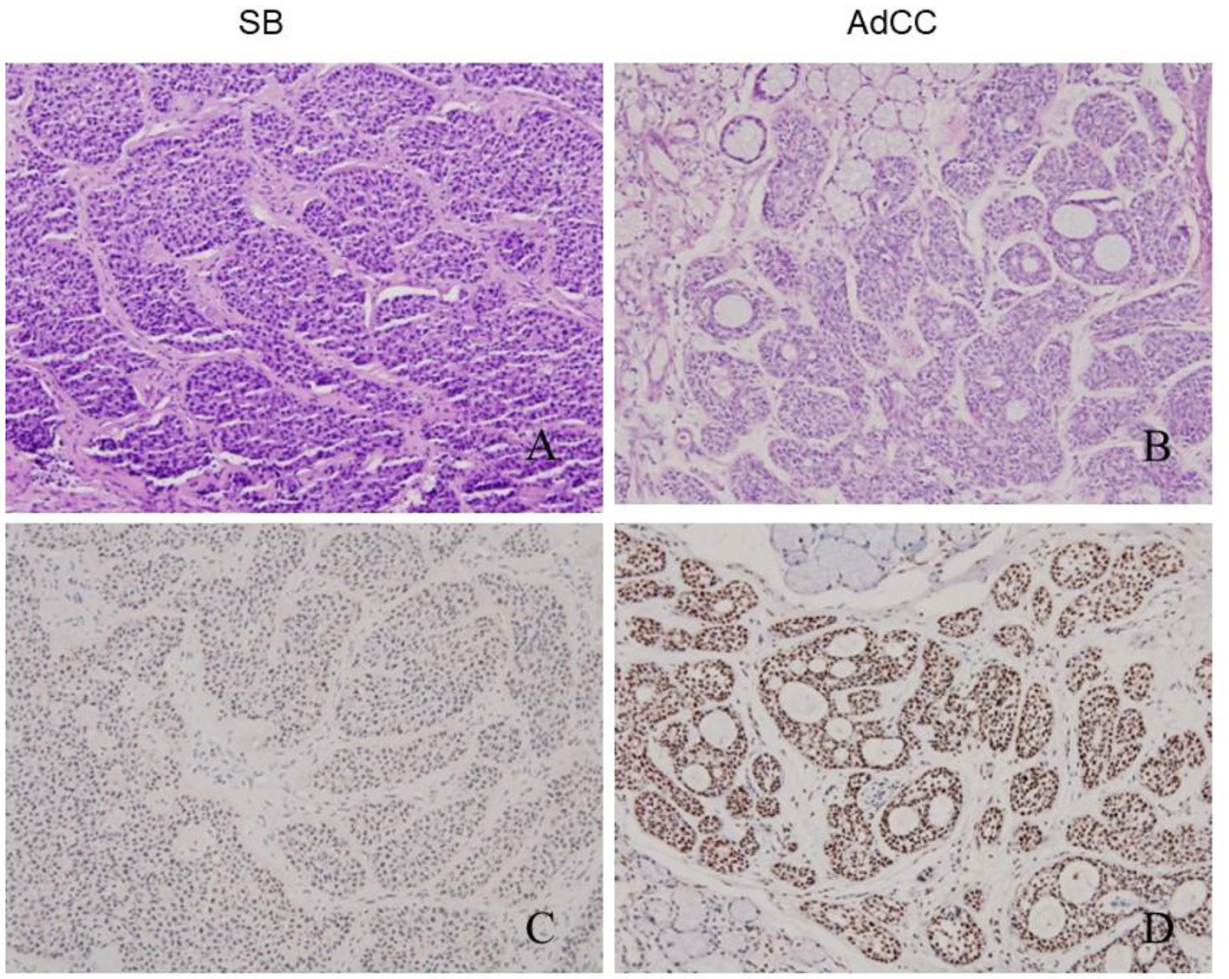
Histology (upper line) and MYB immune-expression (lower line) in SB and AdCC. SB composed of basaloid epithelial nests with a palisading pattern **(A)**. AdCCs in this study were all typically cribriform, and exhibited invasion into normal gland **(B)**. MYB immunostains were strongly and diffusely positive in myoepithelial cells and partial glandular tumor cells of two SBs **(C)** and all the AdCC in this study **(D)**.

SB showed nests of basaloid cells with a palisading pattern at the periphery, and maturation toward the center. Neither brisk mitotic activity nor necrosis was present (Figure 2A). The characteristic morphology and specific age of onset of this tumor were the key points of diagnosis.

In contrast, the AdCCs under study were all typically cribriform, and exhibited invasion such as that into muscle, gland, nerve, or bone. Multiple microcytic spaces divided the nests into numerous cylinders, yielding a “honeycomb” configuration (Figure 2B). The nuclei were atypical and appeared hyperchromatic and pleomorphic with increased mitotic activity. The stroma in AdCC appeared more hyalinized and richer than other lesions noted above. Moreover, the separation between the stroma and parenchyma was obvious.

### Cytomorphologic Descriptions

Although cancer cells in AdCC were in some instances more pleomorphic and hyperchromatic than cells in benign diseases, cytologic features alone did not serve to distinguish AdCC from its mimics. All the mimics showed overlapping cytological features with AdCC. The nuclei of the mimics were regular in shape and uniformly basophilic. Ductal structures were lined by cuboidal cells which had uniform nuclei with condensed chromatin. An inner layer of cuboidal cells was surrounded by the outer layer of cells, which exhibited myoepithelial differentiation. Nuclear atypia was absent or minimal, and the mitotic figures were rare.

### Immunohistochemistry

The overall results are listed in Table 2. iBCA, cBCA, and IDH showed no MYB antigen immunoreactivity (Figures 1D–F). However, two cases of SB were MYB positive (Figure 2C). As a contrast, all AdCC exhibited strong diffuse nuclear expression of MYB in myoepithelial and partial glandular tumor cells (Figure 2D).

**Table 2:** Immunohistochemical and FISH results of all the basaloid lesions.

**Tumor**	**Myb**	**βcatenin**	**CD117**	**SOX10**	**ki67 (%)**	**P63**	**calponin**	**FISH-MYB**
cBCA	-	+(including nuclear)	+(5/23)	+	0.30%	+	+	-
iBCA	-	+(including nuclear)	+(3/11)	+	2.80%	+	+	-
SB	+	+	+(3/3)	+	37.30%	+	+	-
IDH	-	+	-	+	1.00%	+	+	-
AdCC	+	+(only membrane)	+(14/20)	+	28.25%	+	+	+

Immunomarkers of p63 disclosed the presence of both basaloid and myoepithelial differentiation, while calponin marker indicated only myoepithelial differentiation here. Most tumor cells in all cases were p63-positive in parenchyma. The calponin antigen was detected in the polygonal myoepithelia and was especially prominent in the outer layer of cribriforming.

**Figure 3:**
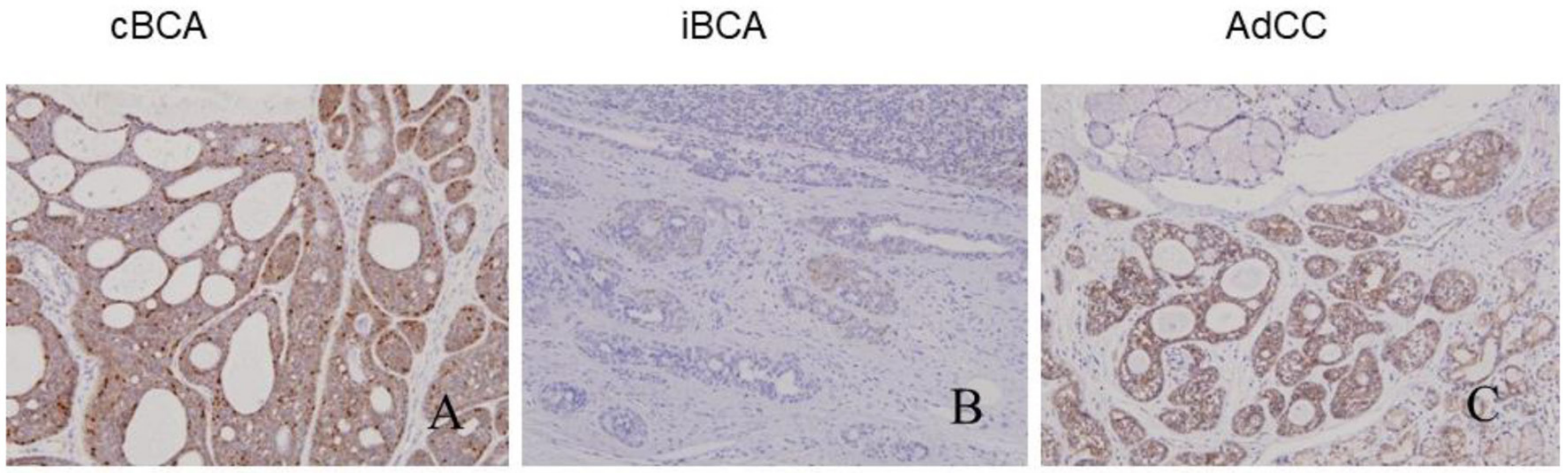
βcatenin immune-expression in cBCA, iBCA, and AdCC. The positive staining of iBCA and cBCA localized in nuclear+membrane or nuclear+cytoplasm+membrane **(A,B)**. AdCC had immuno-activity of β-catenin only in the membrane **(C)**.

All tumors showed varied patterns of β-catenin expression: positive iBCA and cBCA staining localized in the nuclei and membrane (7 iBCA and 17cBCA), or nuclear+cytoplasm+membrane staining (4 iBCA and 6 cBCA). However, AdCC showed β-catenin immunoreactivity only in the membrane. Two cases of SB showed positivity only in the membrane, and in one case there was nuclear+cytoplasm+membrane positivity. Four cases of IDH were found to be nuclear+membrane positive, while three cases of IDH showed positivity solely in the membrane (Figure 3).

CD117 generally showed a positive trabecular pattern. Five cases of iBCA and three cases of cBCA were positive for CD117. Two cases of SB were positive for CD117. IDH was CD117 negative. CD117 was expressed in 14 cases of AdCC. Sox10 was expressed in all cases.

The average expression ratio of ki67 in cBCA, iBCA, SB, IDH, and AdCC was 0.3, 2.8, 37.3, 1.0, and 28.25%, respectively. The ki67 indices of SB and AdCC were significantly higher than that of cBCA, iBCA, and IDH (*p* < 0.05, *p* < 0.05, *p* < 0.05, respectively). The ki67 positive index in SB and AdCC was not significantly different.

### Evaluation of *Myb* Rearrangement by FISH

**Figure 4:**
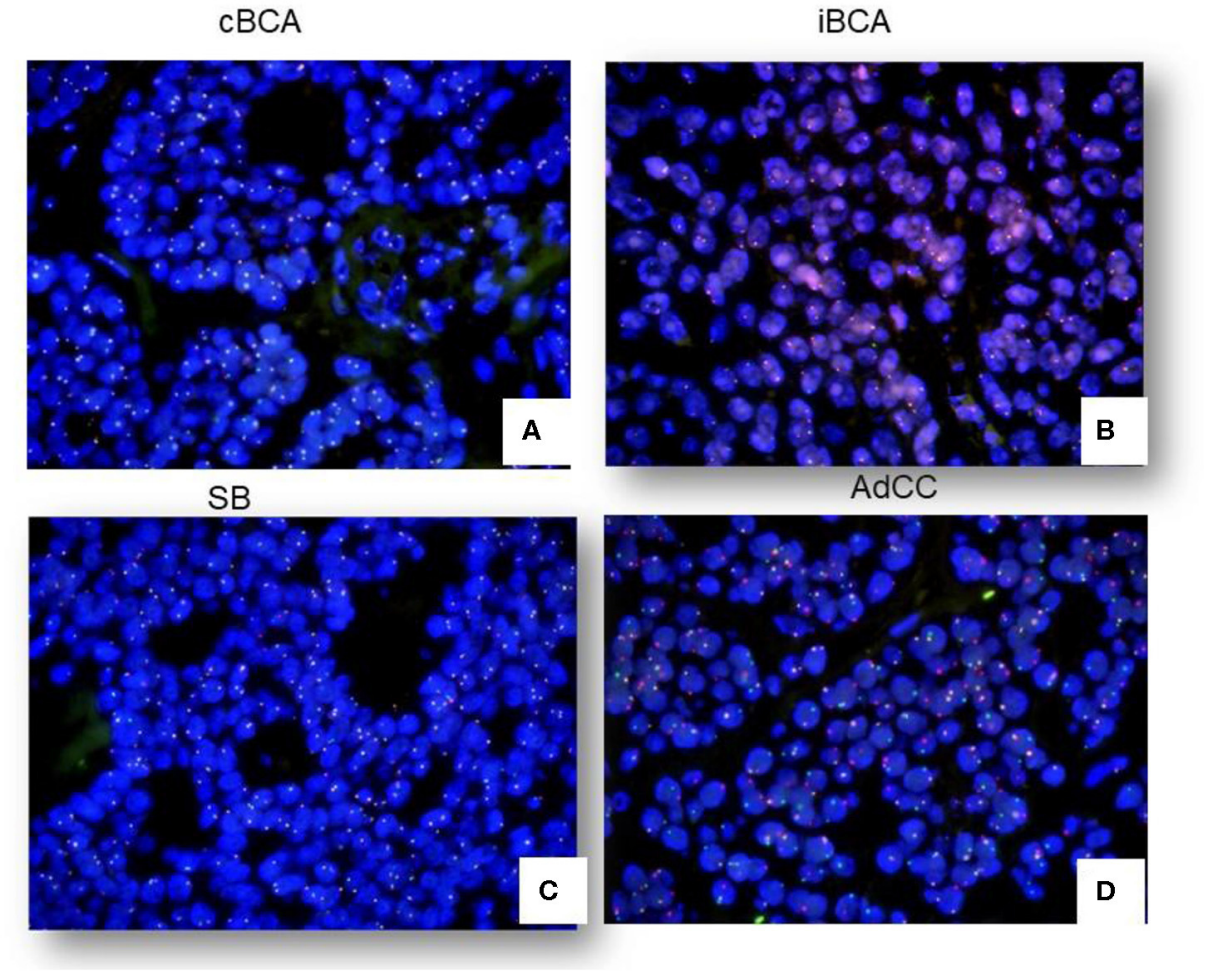
FISH signals in cBCA, iBCA, SB, and AdCC. No cases of cBCA, iBCA, or SB showed Myb rearrangement **(A–C)**. 73.68% of AdCC showed positive signals by FISH **(D)**.

None of these lesions under study, including cBCA, iBCA, IDH, and SB, showed *Myb* rearrangement by FISH. One case of cBCA and one case of iBCA did not yield enough evaluable signals due to long storage of blocks and poor probe penetration of nuclei (Figures 4A–C). Fourteen cases of 19 AdCC (73.68%) were found to harbor Myb rearrangement (Figure 4D). One case of AdCC showed no signal due to poor specimen preparation.

Noticeably, the MYB immunoprofile was significantly related to FISH results (*p* = 0.00, Pearson correlation).

## Discussion

Morphologic studies of salivary basaloid lesions have of late received attention (13). This study focused on the differential diagnosis of AdCC and basaloid lesions which mimic AdCC. Our data suggested that an immunoprofile incorporating MYB, ki67, and β-catenin together with FISH was advantageous for diagnosis, and these markers should be considered as first line markers. Alternative markers, including CD117, SOX10, p63, and calponin, were not as specific as MYB, ki67, and β-catenin.

Although AdCC grows comparatively slowly, it should be considered as at least a moderately differentiated cancer because of its predilection for perineural invasion, local recurrence, and distant metastasis even many years after the initial surgery (14). Therefore, its overall prognosis is poor. The recurrent *t* (6; 9) (q22-23; p23-24) translocation has been identified in AdCC (15), resulting in scrutiny of Myb in the diagnosis of AdCC. The poor prognosis of this tumor may be associated with MYB expression (16). However, the MYB expression showed no correlation with traditional prognostic factors such as TNM stage or tumor grade (17). As for the location in which MYB is expressed, it has been reported that MYB immunolabeling is confined mainly in the peripheral myoepithelial cells and has not been identified in ductal cells of tumors with either a tubular or cribriform pattern (16). We chose typical cribriform AdCC as a control and found that these tumors all showed strong MYB expression in both myoepithelial and partial glandular tumor cells. There were also controversies in the choice of immunohistochemistry and FISH to evaluate for MYB antigen or fusion of Myb and NFIB. Poling et al. found that MYB-immunohistochemistry was more sensitive and specific than Myb-FISH (18). Using the FISH technique, West et al. found a balanced translocation between Myb and NFIB was present in 49% of AdCC, while there was no apparent translocation of *Myb* in 35% of the cases (16). Sixteen percent of cases have an abnormal FISH pattern suggestive of a translocation of Myb but not necessarily involving NFIB (Myb 3′, Myb 5′, and NFIB all come together, extra 5′Myb without association with NFIB, and Myb split without association with NFIB). In our study, MYB expression was not observed in iBCA, cBCA, or IDH. MYB immunostain appeared to be a sensitive marker for differentiating AdCC from salivary gland lesions with a similar histology, except SB. However, the positive-MYB SB in immunohistochemistry showed no Myb rearrangement by FISH, while 73.68% of AdCC were found to harbor a Myb rearrangement. This strongly suggested that MYB immunostaining is a specific and cost-effective method in the diagnosis of AdCC, but further confirmation by FISH was recommended.

SB is an extremely rare tumor, and <100 cases have been reported worldwide (19). In most of the reported cases, SB occurred in the parotid, and these cases had all been diagnosed at birth. Our cases of SB involved the parotid gland, but the age of onset was a little older than infancy. There were a few youth cases of 13-years-olds reported in literature (20). The histologic features of these cases were microscopically similar to those in gland embryonic development at the 3rd month. Some pathologists believe IDH is the precursor of BCA. In the current study we found that MYB immuno-expression and the ki67 index overlapped with AdCC. As Myb is the key finding in the diagnosis of AdCC, it is critical to ensure diagnosis by FISH. None of the tumors evaluated in our study showed evidence of fusion of Myb and NFIB by FISH, except AdCC. 73.68% of AdCC showed Myb-NFIB fusion. There was a significant positive correlation of the MYB immunoprofile and Myb rearrangement. As such, we conclude FISH is a reliable diagnostic approach for differentiating basaloid lesions from AdCC.

β-catenin was useful in this study for distinguishing BCA from AdCC, including cBCA and iBCA. Its nuclear expression was typical and specific for BCA. Sato et al. demonstrated that Wnt/β-catenin signal alteration plays a role in the pathogenesis of BCA (4). Our data showed that all the selected AdCCs in our study had membrane immuno-activity of β-catenin. One case of SB was nuclear+cytoplasm+membrane positive, and four cases of IDH were nuclear+membrane positive. The above situations are infrequent but should be kept in mind in differential diagnosis.

Ki67 is widely used as a proliferation marker (21). Generally, benign neoplasms have lower ki67 indices, typically <10%. Although the average positive rates of ki67 in SB and AdCC were 37.3 and 28.25%, respectively, there was no statistic difference between them. This may serve as a reminder that the ki67 index should be taken into consideration in order to make an accurate diagnosis.

CD117 expression in AdCC varies considerably. As a proto-oncogene encoding a transmembrane receptor type tyrosine kinase, early reports suggested that CD117 expression appeared to be restricted to AdCC (22). However, our present study found that it may be expressed in most ductal cells, even including benign salivary gland diseases such as most iBCA, and a small number of cBCA and SB. IDH showed no reactivity to CD117. Therefore, CD117 was of little value in the diagnosis of AdCC. SOX-10 was all positive in our series of cases. Although SOX-10 is a marker for sero-mucinous differentiation (23), our findings demonstrated that it is not a specific marker for AdCC.

Taken together, we first used a combination of histologic, immunohistochemical and FISH test to evaluate MYB, CD117, SOX10, βcatenin, ki67, p63, and calponin in the differential diagnosis of cBCA, iBCA, SB, and IDH from AdCC. Considering their sensitivity and specificity, FISH-Myb and an immunohistochemical panel of MYB/β-catenin/ki67 would be an optimal choice for the differential diagnosis of these basaloid lesions.

## Data Availability Statement

All datasets generated for this study are included in the article/supplementary material.

## Ethics Statement

The studies involving human participants were reviewed and approved by Peking University School and Hospital of Stomatology. Written informed consent for participation was not required for this study in accordance with the national legislation and the institutional requirements.

## Author Contributions

BL: design, IHC test, and writing. WJ: follow-up and data analyse. HH: FISH, writing, and submission. All authors contributed to the article and approved the submitted version.

## Conflict of Interest

The authors declare that the research was conducted in the absence of any commercial or financial relationships that could be construed as a potential conflict of interest.
